# Supplementary material for: Mapping the evidence for patient and public involvement and engagement in statistical methodology research: A scoping review protocol
Source: PLoS One. 2025 Dec 8;20(12):e0330232. doi: 10.1371/journal.pone.0330232 (PMC12685195; doi:10.1371/journal.pone.0330232)
Supplement: S2 Appendix — (DOCX) [file pone.0330232.s002.docx]

**Search strategy**

**High sensitivity patient and public involvement search filter** [1]

| 1 | Community Participation/ |
| --- | --- |
| 2 | Patient Participation/ |
| 3 | 1 or 2 |
| 4 | (patient* or public or lay or people or consumer* or user* or citizen* or parent or parents or child*).ti,ab. |
| 5 | (participat* or involv* or engag* or consult* or collaborate* or conducting or conducted or contrib*).ti,ab. |
| 6 | (questionnaire* or interview* or focus group* or workshop* or peer led or research or self-report* or qualitative or patient led or public led or self rating or self rated or development).ti,ab. |
| 7 | 4 and 5 and 6 |
| 8 | ((health or research) and (partners or partnership)).ti,ab. |
| 9 | 3 or 7 or 8 |

**MEDLINE (Ovid) Search**

| 1 | Community Participation/ |
| --- | --- |
| 2 | Patient Participation/ |
| 3 | 1 or 2 |
| 4 | (patient* or public or lay or people or consumer* or user* or citizen* or parent or parents or child* or carer*).ti,ab. |
| 5 | (participat* or involv* or engag* or consult* or collaborate* or conducting or conducted or contrib* or co-produc* or co-develop* or co-creat*).ti,ab. |
| 6 | (questionnaire* or interview* or focus group* or workshop* or peer led or research or self-report* or qualitative or patient led or public led or self rating or self rated or development).ti,ab. |
| 7 | 4 and 5 and 6 |
| 8 | ((health or research) and (partners or partnership)).ti,ab. |
| 9 | 3 or 7 or 8 |
| 10 | (statistic* methodolog* or statistic* research or methodolog* research or trial* methodolog* or model* methodolog* or data science).ti,ab. |
| 11 | 9 and 10 |
| 12 | limit 11 to yr=”1996-current” |

**Google, DuckDuckGo and Mednar search**

| **PPIE terms** | **Statistical methodology terms** |
| --- | --- |
| community participation | statistic methodolog |
| co-production | statistic research |
| patient engagement | methodolog research |
| patient participation | trial methodolog |
| PPI | model methodolog |
|  | data science |

Searches will be conducted for all combinations of terms from column 1 and column 2 as follows:

| community participation statistic methodolog |
| --- |
| community participation statistic research |
| community participation methodolog research |
| community participation trial methodolog |
| community participation model methodolog |
| community participation data science |
| co-production statistic methodolog |
| co-production statistic research |
| co-production methodolog research |
| co-production trial methodolog |
| co-production model methodolog |
| co-production data science |
| patient engagement statistic methodolog |
| patient engagement statistic research |
| patient engagement methodolog research |
| patient engagement trial methodolog |
| patient engagement model methodolog |
| patient engagement data science |
| patient participation statistic methodolog |
| patient participation statistic research |
| patient participation methodolog research |
| patient participation trial methodolog |
| patient participation model methodolog |
| patient participation data science |
| PPI statistic methodolog |
| PPI statistic research |
| PPI methodolog research |
| PPI trial methodolog |
| PPI model methodolog |
| PPI data science |

**References**

[1] M. Rogers, A. Bethel, and K. Boddy, "Development and testing of a medline search filter for identifying patient and public involvement in health research," *Health Information & Libraries Journal,* vol. 34, no. 2, pp. 125-133, 2017, doi: <https://doi.org/10.1111/hir.12157>.
