# Supplementary material for: Mapping the evidence for patient and public involvement and engagement in statistical methodology research: A scoping review protocol
Source: PLoS One. 2025 Dec 8;20(12):e0330232. doi: 10.1371/journal.pone.0330232 (PMC12685195; doi:10.1371/journal.pone.0330232)
Supplement: Attachment — Submitted filename: Response to Reviewer.docx [file pone.0330232.s004.docx]

Dear reviewer,

Thank you for your thoughtful comments on this scoping review protocol that we have addressed below. All line locations refer to the track changes version of the manuscript document.

*1. It is unfortunate that this protocol is being published after the study has already commenced in July and is expected to be completed by September (this month). This timing reduces the function of the paper as a tool for transparency and pre-study registration, and also limits the opportunity for review comments to help strengthen the work. I did not find information on whether the protocol was published elsewhere before the start of the study; This should be clarified. The timing of the protocol submission should also be made clear in the manuscript. Nevertheless, I provide my comments below.*

The authors would like to apologise, the dates given in the submitted protocol are not accurate. The work on the review began in August 2025, we estimate that the review will be complete by early 2026. At the time of responding, there is still the opportunity for the reviewer’s comments to be incorporated. The timeline of the review has been edited to reflect this (lines 131-134). The protocol was not registered/published anywhere else before the start of the review.

*2. Regarding inclusion: Is patient and public involvement in the design of applied studies considered eligible? For instance, if PPI is integrated at all stages of an empirical health related study, including design, and the experiences are documented, would such a paper qualify for inclusion, even if it is not a methodological paper on its own? If a paper includes a patient or lay person as co-author, but does not explicitly describe their involvement, will it still be considered for inclusion?*

Our study is focussed on PPI for methodological research. For your example given, this would not have met our inclusion criteria if the PPI was concerned with advising on the methods for a study (e.g. choosing the preferred study design from a list of existing options). For a study to meet our criteria, we would require the PPI to be concerned with methodological research (e.g. development of novel study methodology). We have expanded the exclusion criteria section to offer further clarification for the reader.

*3. When discussing quantitative healthcare methodological research, the authors mention clinical trials, health data science, mathematical modeling, and simulation studies. Does this also include the design of observational cohort studies, case studies, or the statistical design of analyses for qualitative studies?*

For all of the study types mentioned we are only interested in PPI for methodological studies. Therefore, if a methodological study was developing, evaluating, or comparing methodological approaches for observational studies and presented information on the PPI conducted these would be included. Studies focussed on qualitative methods are excluded, although studies which use qualitative methods to assess PPI in quantitative methodological studies would be included. We have amended the context section (line 160-163) to make this clearer.

*4. The authors intend to include grey literature, but the protocol lacks detail on the standards, limitations, or formats applied to this category. For example, would a folder, a video recording, or an opinion blog be eligible? What would not be?*

We would include any data source as long as there was sufficient information present to extract data around the “key themes” of the scoping review (lines 274-281). We have added a sentence to this effect in the manuscript (lines 230-232).

*5. Based on these points, I feel the methodology is not yet explicit enough to guarantee transparency or support replication. Greater detail should be provided on study inclusion and exclusion criteria and the reasons for these decisions. A PICOS framework could help structure this, as could examples of papers that would qualify for inclusion.*

We have been following the JBI Manual for Scoping reviews (<https://jbi-global-wiki.refined.site/space/MANUAL/355862707/10.2.4+Inclusion+criteria>) where the PCC framework is used (Population, Concept, Context) rather than the PICOS framework. Line 128 has been edited to add this detail and we have added a ‘population’ subsection in the inclusion criteria section (lines 139-140).

We have also given an example of a paper that we were aware of from our initial scoping of the literature (before the systematic searches began) (lines 190-193)

*6. The authors restrict the search to papers published after 1996, when the advisory group on consumer involvement was established, describing this as a milestone in modern PPIE. However, they do not explain why earlier studies are considered irrelevant. Why does this milestone justify excluding earlier literature?*

We have edited this (line 173-174) as the choice of phrasing was perhaps unclear. We chose the cut off-of 1996 as we judge this to be the beginning of PPIE (although not called that at the time). To date, we have not found any papers published between 1996 and 2014 that meet our inclusion criteria, so we think it is highly unlikely that there is any relevant literature prior to 1996.

*7. The authors have limited grey literature sources to the first 20 results. What is the rationale for this cutoff? Do they have supporting evidence? For comparison, Wichor Bramer et al. examined the yield of additional references within the first 100–200 results of Google Scholar searches, and their findings could serve as guidance for setting such a limit.*

The rationale for this cutoff is given in lines 227-228 (“This will result in many searches, therefore, we will restrict our findings to the first 20 results to maximise search efficiency.”). Due to the many ways that authors may phrase both “statistical methodology” and “PPIE”, we have chosen to adopt a “broad and shallow” grey literature search strategy consisting of 30 different key phrase combinations across 3 different search engines (so 90 searches in total) with the aim of finding as much literature as possible using these key phrases. It would be very time consuming to screen between 9,000 and 18,000 search engine results and, unfortunately, we don’t have the resources for this. We have changed the discussion section (lines 316-319) to acknowledge this is a limitation.

*8. On page 9, line 234, the authors state they “anticipate” that the data extraction form will capture certain information. In the context of a review protocol, however, data items to be extracted should be prespecified (as required in registries such as PROSPERO). Any additional extraction should later be reported as deviations from the original protocol. Simply “anticipating” extraction items risks later omission of items that yield unfavorable results, which could introduce bias or raise concerns of cherry-picking. Prespecification is therefore an important transparency safeguard.*

The data extraction section has been edited to remove the word anticipate so it is clear that we **will** extract these data. We have also done further edits in this section to explain that the structured and narrative data extraction will take place using two different platforms.

*9. On page 10, under patient and public involvement: the PPIE group is described as advising the research group. Given the nature of this study, it would be helpful to clarify the extent of their role. Are they solely advisory (with recommendations that may be ignored), or are they members of the research team? Do they contribute to manuscript drafting and revision, or do they remain external advisors?*

We have clarified this section further. We have a PPI co-applicant (Samina Begum) who is part of the research team. Samina inputted to the design of the research at the application stage and has now reviewed the manuscript and has been added to the author list. Samina chairs our public advisory group which has 6 members, this group will advise the research team and meet every 6 months. Both Samina and the PAG will attend the externally facilitated meeting to review the results and develop the visual evidence map.

*10. I disagree with the statement under PRISMA-P that risk of bias is “not applicable.” Bias can arise in all forms of research, whether qualitative or quantitative, systematic or scoping. The authors should reflect on the possible sources of bias and limitations in this review, including when narrative synthesis is applied. For example, are included studies written by groups with a vested interest?*

We have now included that we will use the CASP Qualitative Methodology checklist as a framework for assessing the risk of bias (lines 262-263) and reflect on potential meta-bias in the discussion section (lines 282-283)

*11. In the data collection framework, I would encourage the inclusion of two topics frequently discussed in patient communities: (1) remuneration and acknowledgment of patient contributions, and (2) diversity of representation within PPI. Capturing such information would add value to the scientific community.*

Thank you for this suggestion. We agree that these topics are important and have included them within the data extraction section (lines 260-261)
